# Focused-Ion-Beam Induced Rayleigh-Plateau Instability for Diversiform Suspended Nanostructure Fabrication

**DOI:** 10.1038/srep08236

**Published:** 2015-02-04

**Authors:** Can Li, Lurui Zhao, Yifei Mao, Wengang Wu, Jun Xu

**Affiliations:** 1 National Key Laboratory of Science and Technology on Micro/Nano Fabrication, Institute of Microelectronics, Peking University, Beijing 100871, China; 2 Electron Microscopy Laboratory, Peking University, Beijing 100871, China

## Abstract

A novel method for fabricating diversiform suspended nanostructures is reported. The method utilizes focused-ion-beam (FIB) induced material redistribution and Rayleigh-Plateau instability, which determine the resulting shapes of formed nanostructures. By choosing target materials, their predefined patterns as well as FIB settings, we have achieved parallel nanofabrication of various kinds including nanostrings, nanobead chains and nanopore membranes with smooth surfaces due to the self-perfection effect of the material redistribution upon the minimization of system free energy. The diameters of the nanostrings and nanopores reach about 10 nm and 200 nm, respectively. The average period of the nanobead chains is 250 nm.

Focused ion beam (FIB) is widely used in solid-state integrated circuit failure analysis^1^,^2^ and modification^3^,^4^,^5^. Recently, it also finds applications for high-precision machining in micro/nanoelectromechanical systems (MEMS/NEMS). Previously reported FIB processes include FIB-induced material sputtering^6^, chemical vapor deposition^7^, and three-dimensional assembly due to introduced uncompensated stress^8^,^9^. Other FIB-induced effects also have potential to be employed as micro-/nano-fabrication methods.

It has been reported that FIB will initiate the surface atom diffusion and it is employed to modify the surface morphology. Lian et al^10^ presented that, under FIB irradiation, the patterned Co lines, concentrate rings and squares on Si substrate were transformed into periodic nanodots due to FIB-induced dewetting and Rayleigh instability. Zhao et al^11^ studied the surface morphology change during the transformation from Au or Pt lines into periodic nanodots on SiO_2_ substrate by FIB irradiation. Naik et al^12^,^13^ further investigated such transformation with Au nanowires on different types of Si, SiO_2_ and Si_3_N_4_, and discussed that the properties of substrates, such as wettability and electrical conductivity, play important roles in the transformation. The process is understood that, under the ion beam bombardment, the target atom mobility increases due to the energy exchange between the incident ion and the target material. The high mobility material atoms redistribute to minimize the system free energy, and thus causes two effects: 1. Series of nanostructures in a certain spatial period formed due to Rayleigh-Plateau instability^14^; 2. Self-perfection to smooth out the structure surface. Similar effects induced by laser^15^,^16^,^17^,^18^ and thermal annealing^19^,^20^ were also reported and found their applications in many nanodevices, e.g. mechanical resonators^21^, optical waveguide^22^, and electronic devices^23^. Even so, FIB has many advantages that other technologies cannot achieve, e.g. high-precision localized irradiation and easily tuning of beam energy and flux.

In this paper, we show our study using FIB to modify and smooth out surface morphology in suspended nanostructures with various materials and predefined patterns. First of all, all the previous papers reported the phenomena for metal materials (Co^10^, Pt^11^, Au^11^,^12^,^13^). Our paper reports the process with different types of materials including crystal/poly-crystal Si, amorphous SiO_2_ and Si_3_N_4_, and metal Au. Secondly, the previous reports focused on the structures on various substrates, while we here report suspended structures. From mechanism aspect, the morphological evolution of suspended structures is more governed by the Rayleigh-Plateau instability, while supported structures also need to consider the properties (conductivity, wettability, etc.)^13^ of the substrates. For the obtained fabrication results, the final substrate-supported structures are mostly periodic dots, while in this paper our final structures are nanostrings, linked nanobead chains, nanopore nets and so on. Thirdly, we study the control of the Rayleigh-Plateau instability by providing a head-start of the instability with predefined patterns, which are not reported in any of the previous paper. Due to the differences in materials, structure suspension and predefined patterns, we can fabricate cleaner structures than those in the previous papers^10^,^11^,^12^,^13^.

With this method, we are able to fabricate predictable nanostructures beyond the machine resolution for some initial patterns we made before large-area irradiation. As demonstration, we have successfully fabricated various suspended ultrafine structures with smooth surface. With different FIB settings, target material types and predefined patterns, we experimentally demonstrate a few typical structures fabricated on suspended film, e.g. nanostrings (less than 10 nm in diameter), fusiform masses (sub-10 nm diameter links), nanobead chains (tens-nm diameter links between nanobeads and average period of 250 nm), and large-area nanopore nets (on Si membranes with average pore diameter in about 200 nm). We believe that it is a critical fabrication technology for many applications including chem-/bio-sensors based on fusiform nanowire resonators^24^,^25^, and fluidic diodes/transistors based on nanopore membranes^25^,^26^, etc.

## Results

### Phenomena

Experiments were carried out on suspended films of various materials including SiO_2_, Si_3_N_4_, poly-/crystal-Si, Au and Al with thickness ranging from 50 nm to 150 nm. An FEI Strata DB235 FIB/SEM dual beam system with Ga^+^ ion source was used, for both predefined nanostructure (such as nano-cantilever) patterning and afterwards energetic ion treatment, which induces structure morphological change.

**Figure 1:**
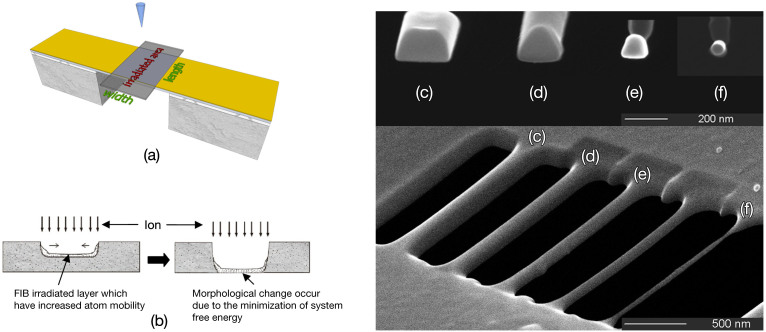
Schematic diagrams of (a) FIB process mode and (b) FIB treatment process. SEM images of morphological evolution from Si cantilever to cylindrical nanostring: (c) without ion irradiation; (d–f) with uniform ion irradiation at fluence of (d) 4.05 × 10^16^ ion·cm^−2^, (e) 1.12 × 10^17^ ion·cm^−2^, (f) 1.52 × 10^17^ ion·cm^−2^, respectively.

To investigate the ion-beam-induced structure morphological change, clamped-clamped nano-cantilevers were made from suspended films by FIB direct milling. They were then uniformly irradiated under a large-area scanning of FIB, as schematically shown in Figure 1a and 1b. Figures 1c–1f show the SEM images of the morphological evolution from a Si cantilever to a nanostring under 30 keV Ga^+^ ion irradiation. The cross-section shape of the structure evolves from trapezoid to circle. The images show that, before large-area ion irradiation (Figure 1c), the cross-section shape, as expected after FIB direct patterning, is near trapezoid. The sloping sides might be generated by the Gauss distribution of the 30 keV Ga^+^ ion beam with a spot size about 23 nm, the amorphization and in some sense liquefaction of the irradiated part, and the redeposition of some sputtered materials. At the first stage of evolution, as is seen from Figure 1d, the cantilever shrinks from the both sides since the ions with larger incident angle have higher sputtering yield^27^. With increased ion irradiation dose, the structure shrinks almost isotropically, and its cross-section shape gradually evolves into semi-circle with unaffected flat bottom (Figure 1e). Finally, after the bombardment dose reached about 1.52 × 10^17^ ion · cm^−2^, the cantilever evolves into a cylindrical nanostring with round cross section (Figure 1f). The diameter of the cross section is measured as approximately 50 nm. The final round cross section indicates that, with the thickness below a critical value, apparent material redistribution occurs upon the minimization of system free energy during the ion beam treatment process.

### Mechanism

It is useful to analyze the interaction of energetic ions with target materials at atom scale in the FIB irradiation. During the interaction, enormous kinetic energy will be transferred to the target atoms (especially since Ga^+^ ion has much larger mass than target atom, such as Si atom). Within tens of picoseconds, the beam causes the displacement of atoms from their crystallographic sites, and further results in the formation of the displacement cascade in the irradiated layer of the material. The displacement cascade will quench, and the defects start to diffuse and the atoms start to redistribute upon the minimization of system free energy in a longer time frame. On the other hand, the ion (such as Ga^+^) bombardment also causes sputtering of the target materials, resulting in gradual decrease of the cantilever thickness. When the thickness reduces to a critical value where the ions are capable of affecting all the materials in the nanostring, all the atoms in the structure therefore start to redistribute. TRIM^28^ simulation shows that the 30 keV Ga^+^ ion implanting into Si has a projected range and longitudinal straggling of 27.8 and 10.3 nm, respectively. The experiment result illustrated in Figure 1f shows that the critical value for whole-structure atom redistribution is about 50 nm, which is larger than the ions projected range. It is because the ions transfer their energy to the target materials through displacement cascade, so that they are capable of affecting larger volume than the projected range. Because the atoms redistributes upon the minimization of system free energy, the structure surface becomes smooth, and we can use the nature of liquid to analyze the surface morphology evolution. In order to simplify the analysis, we regard the structure with thickness reduced to be less than the average projected range of the incident ions as a quasi-liquid nanobridge.

Through the in-situ observation with the SEM imaging ability of the FIB system, we find that the incident-energetic-ion-driven redistribution and surface self-perfection process of the target materials only occurs when the ion beam is on. Considering the FIB-induced “quasi-liquid nanobridge” stage, we can borrow the concept from Rayleigh-Plateau instability to have a better understanding of the redistribution and surface self-perfection process. The characteristic time scale of Rayleigh-Plateau instability of a liquid cylinder can be described as 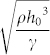
29, where *ρ* is the density of target material in liquid phase, *h*_0_ the radius of the cylinder, and *γ* the surface tension. For a quasi-liquid Si bridge with 30 nm radius, the characteristic time is calculated at hundreds of picoseconds. Since it is longer than the time scale of atom redistribution, we can principally interrupt the redistribution and surface self-perfection process to “freeze” the non-equilibrium state during the morphological evolution.

**Figure 2:**
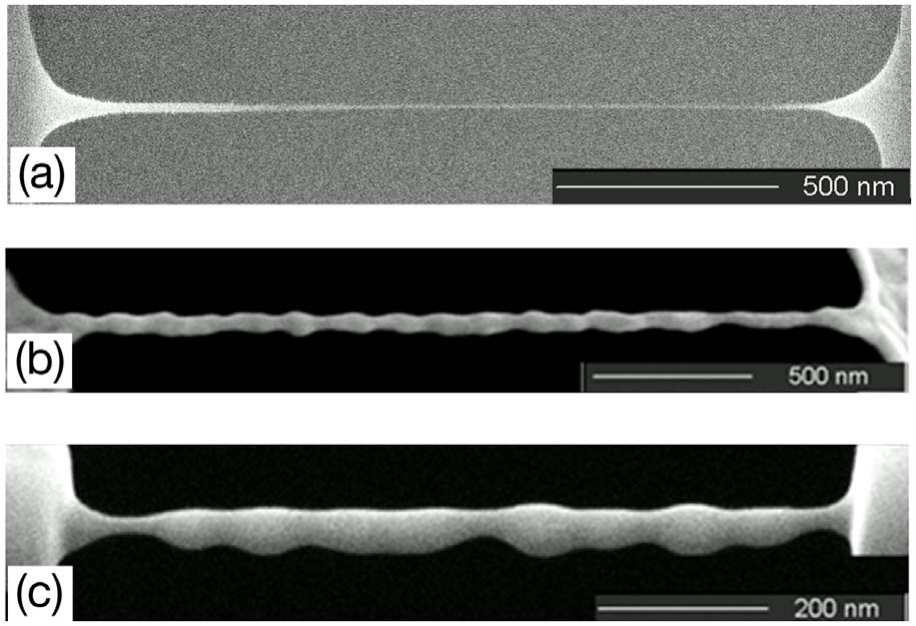
SEM images of some structures obtained by FIB process after quasi-liquid bridges were formed. (a) Uniform sub-10 nm nanostring made of a Si_3_N_4_ cantilever and (b, c) nonuniform shaped nanostructures made of Au and poly-Si cantilevers, respectively.

In general, with further ion irradiation, the morphological evolution will continue according to both the ion-beam-induced sputtering and redistribution of target materials. In our experiments, two phenomena were observed depending on specific materials and FIB settings: uniform nanostructures due to stability of the redistribution and surface self-perfection process, and non-uniform nanostructures due to instability of the redistribution and surface self-perfection process. Figure 2a shows a uniform nanostring obtained on Si_3_N_4_ material. Figures 2b and 2c illustrate non-uniform nanostrings formed on Au and poly-Si materials, respectively. It is noteworthy that all the uniform nanostructures were obtained from amorphous materials (Si_3_N_4_, SiO_2_), while non-uniform nanostructures were obtained from poly- and single-crystalline materials (poly- and single-crystalline Si).

The morphological evolution, resulting in two different modes of stability or instability, is affected by the natures of target materials, and has the trends toward to the minimization of system free energy. Under certain perturbation, the cylindrical nanostring bridge tends to agglomerate into series of beads due to the minimization of surface energy. This is because the final structure morphology to be obtained will be the one with the smallest overall surface area, since the free energy of the system is the product of surface tension and surface area under only consideration of surface tension. Due to the fundamental physical similarity between the surface-energy-driven dewetting and this redistribution and surface self-perfection process, it is reasonable taking the high-energetic atom redistribution stage as a quasi-liquid state to simplify the analysis. Thus, the phenomenon can be analyzed in the Rayleigh-Plateau model^28^, which is based on liquid. This model explains why and how a fluid breaks into series of small liquid drops instead of one single drop which has smaller surface area in the same volume. The model manifests that, before liquid breaking-up, the liquid is disturbed by perturbation waves with various wavelength. The perturbation amplitude decreases when the perturbation wavelength is less than the circumference of an original liquid cylinder (i.e. the system is stable), whereas grows in other cases (i.e. the system is unstable). The perturbation amplitude grows fastest with a certain wavelength (fastest growing wavelength, FGW), and therefore the liquid will finally break into series of drops with this wavelength. According to the model, disturbance, on the other hand, could be diminished due to stretching and internal convection in liquid (e.g. the ion-induced “quasi-liquid nanobridge region” in this work), which requires tensile stress (e.g. fast pulling), and high viscosity (e.g. honey threads are pulled out from tans), respectively. These factors are not considered in linear analysis of Rayleigh-Plateau model and can be ignored for flows with low viscosity and no stress applied. Based on the above mechanisms, uniform nanostrings and nonuniform nanostructures are formed depending on different equivalent material natures under ion irradiation as well as in-time stop of the ion processing.

Consider incompressive, irrotational and inviscid fluid, the Rayleigh-Plateau model based on linear analysis predicts that the structure undulation grows exponentially as the form *a*(*t*) = *a*_0_*e*^−*iωt*^, where *a* is the corresponding amplitude, and *ω* follows the equation that (Equation (1)) 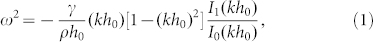
where *k* is the wave number, *h*_0_ is the radius and *I_n_*(*x*) are the Bessel functions of the nth order. From this equation, the perturbation with a wavelength as *λ*_max_ ≈ 9.01*h*_0_ grows fastest in the system.

**Figure 3:**
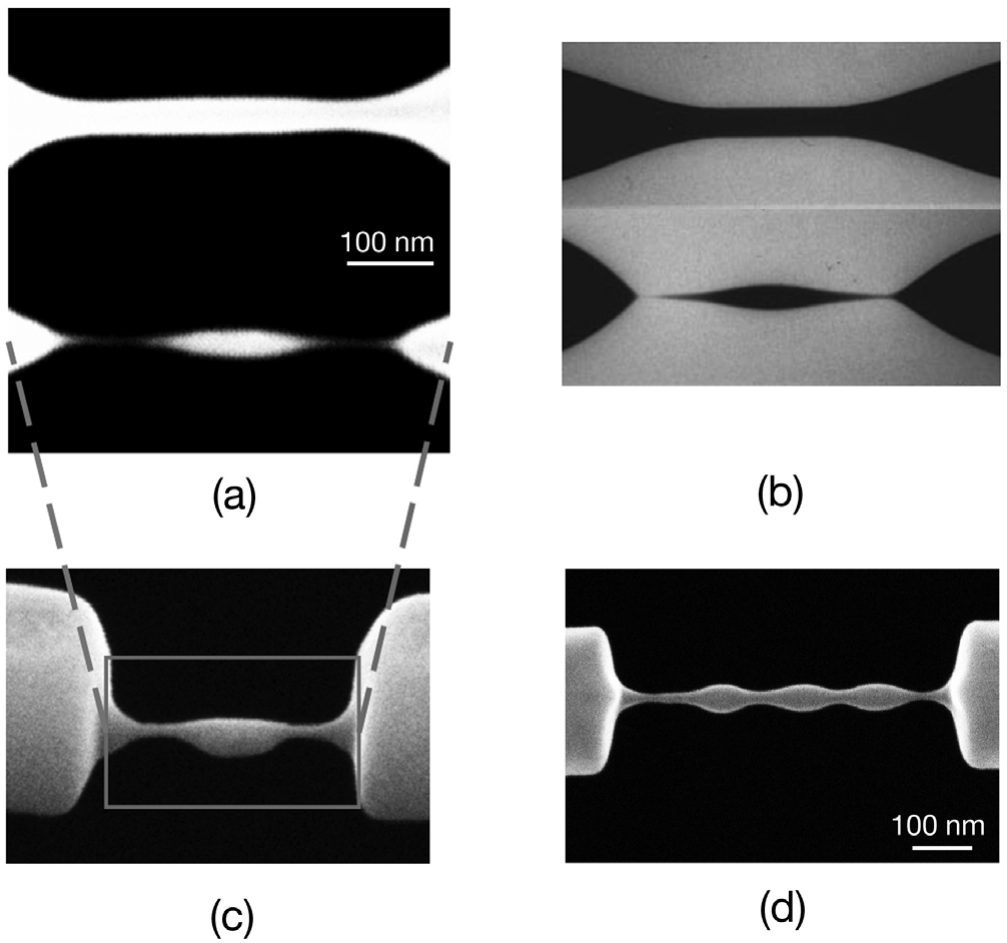
Comparison of (a) a FIB-processed quasi-liquid bridge and (b) a breaking up liquid^24^. (c) Side view of the FIB quasi-liquid bridge taken at 52 degree tilt. (d) Non-uniform “standing-wave” like nanostructure made of a poly-Si clamped-clamped cantilever.

Therefore, a range of perturbation will cause the instability of such a cylindrical liquid bridge, but the average period of the finally obtained structure (before breaking up) should be close to the wavelength of the fastest growing perturbation. We compare the shape of one typical fabricated nanostructure (Figure 3a) due to FIB-induced material redistribution and surface self-perfection with the shape of a breaking-up liquid^29^,^30^ (Figure 3b), and find their shapes are perfectly matched. This phenomenon was observed on different materials include crystal-/poly-Si and Au, which have a fixed melting point and accordingly relative low viscosity in liquid phase. Figure 3d shows the SEM image of a “standing-wave” shaped nanostructure formed due to the instability of a quasi-liquid Si nanobridge with lager length-diameter ratio under FIB irradiation. From the image, the average period of the “standing-wave” structure is measured to be 139.7 nm. The average radius of the agglomerated nanobridge cylinder before breaking up is 14.94 nm. So the period-radius ratio is 9.35, which is consistent with the theory prediction shown above.

### Fabrication methods

#### Predefined patterns

**Figure 4:**
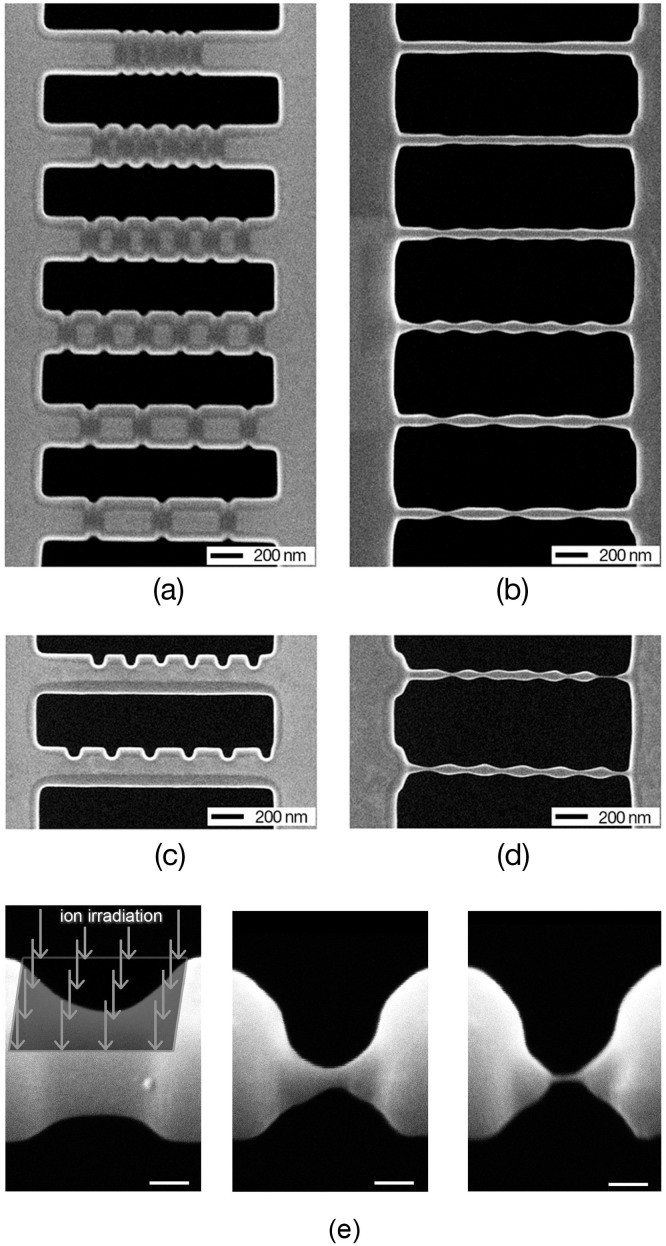
Predefined patterns provide a “head-start” for nanostructure evolution towards instability. (a) The spatial periods of the pre-patterning, from top to bottom, are 100 nm, 150 nm, 200 nm, 250 nm, 350 nm, and 450 nm, respectively. (b) Corresponding nanostructure morphologies got after large-area uniformly FIB irradiation. (c) Asymmetric predefined patterns lead to (d) symmetric structures after ion irradiation. (e) The process of nanostring formation, for material of poly-Si, under the ion beam irradiation which width is much less than the FGW of certain perturbation. The scale bars in (e) represent 50 nm.

As another demonstration of the theory, we also find that predefined patterns on nano-cantilevers can provide a “head-start” for the morphological evolution of the structures, and thus decrease dispersion and result in designed structures with high fidelity to predefined spatial periods in the patterns, especially when the periods match the FGW of certain perturbation. As shown in Figure 4, patterns with different spatial periods were predefined on some crystal-Si nano-cantilevers by FIB direct writing (Figure 4a). The cantilevers were then uniformly irradiated by FIB (Figure 4b). The result reveals: (1) The initial patterns with the spacing much shorter than certain FGW exhibit negative growth rate, which implies that high-spatial-frequency roughness is diminished during the FIB irradiation, and the obtained structures do not follow the predefined patterns; (2) The initial patterns with the spacing approaching to certain FGW lead to perfect sinusoid-shaped nanobeads chains; (3) The initial patterns with the spacing much longer than certain FGW results in the fabrication of fusiform masses linked each other with nanolinks. Noteworthy, the obtained shapes are always symmetric regardless whether the predefined patterns are symmetric or asymmetric, and regardless their undulation looks like sinusoidal, square or other shape waves (Figures 4c and 4d). It is also a clear proof that the Rayleigh-Plateau model plays a key role in the formation of the final morphology.

When the width of irradiation area (Figure 1a) is less than the FGW of perturbation for certain quasi-liquid nanobridge cylinders, skinny uniform nanostrings would be always obtained, regardless FIB settings and target materials. Figure 4e shows the Si nanostring formation process, using the same FIB parameter but different irradiation width with that in Figure 3d. In this situation, only the high-spatial-frequency satellite perturbations with wavelengths much shorter than the FGW exist within the FIB irradiation width, and they are thus diminished during the FIB process, resulting in forming uniform suspended nanostring structures. During the morphology evolutions, as shown in Figure 4e, the shape of the FIB irradiated area is catenoid due to the minimization of overall surface area^31^, which is the nature of liquid.

**Figure 5:**
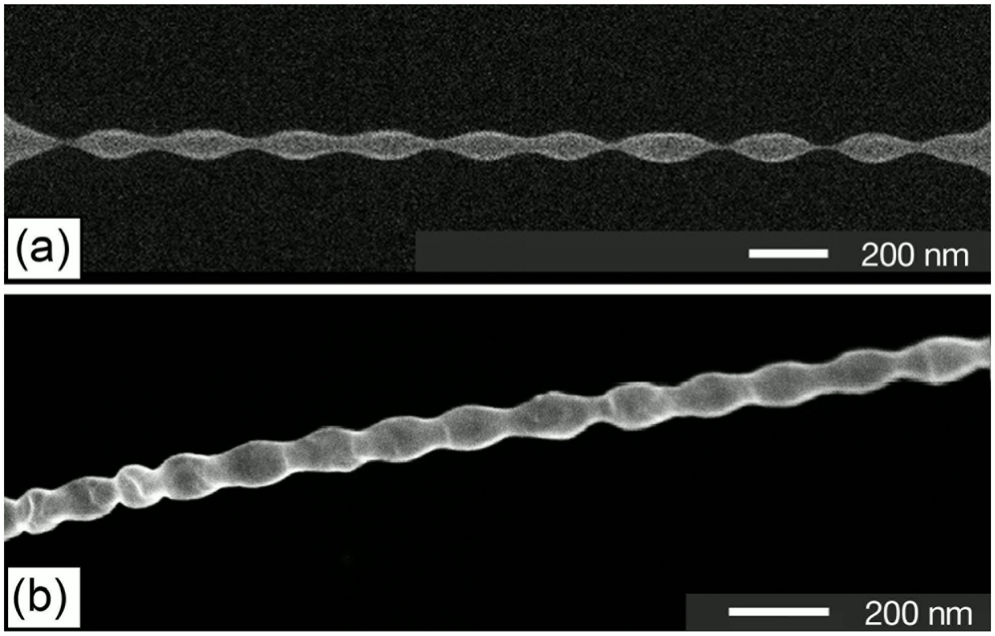
(a) Top view of a nanobead chain in which the beads are linked by tens-nm diameter nanostrings and (b) side view of another nanobead chain, which were fabricated by FIB irradiation after adopting pre-patterning on (a) crystal-Si and (b) poly-Si cantilevers. Both the spatial period are set to be 250 nm.

By employing the above approach, nanobead chains were fabricated (Figure 5) by predefining proper patterns and then exerting uniform FIB irradiation on nano-cantilevers. When the predefined spatial periods fit to the FGW of certain perturbation, as shown in Figures 5a and 5b, some nanobeads are obtained and linked by nanostrings with diameters down to about 10 nm to form a chain. In Figure 5b, there are some obvious irregularities on the formed structure, but there is not in Figure 5a. It is probably due to the poly-crystalline nature of the materials, and irregularities occur on the crystal boundaries.

**Figure 6:**
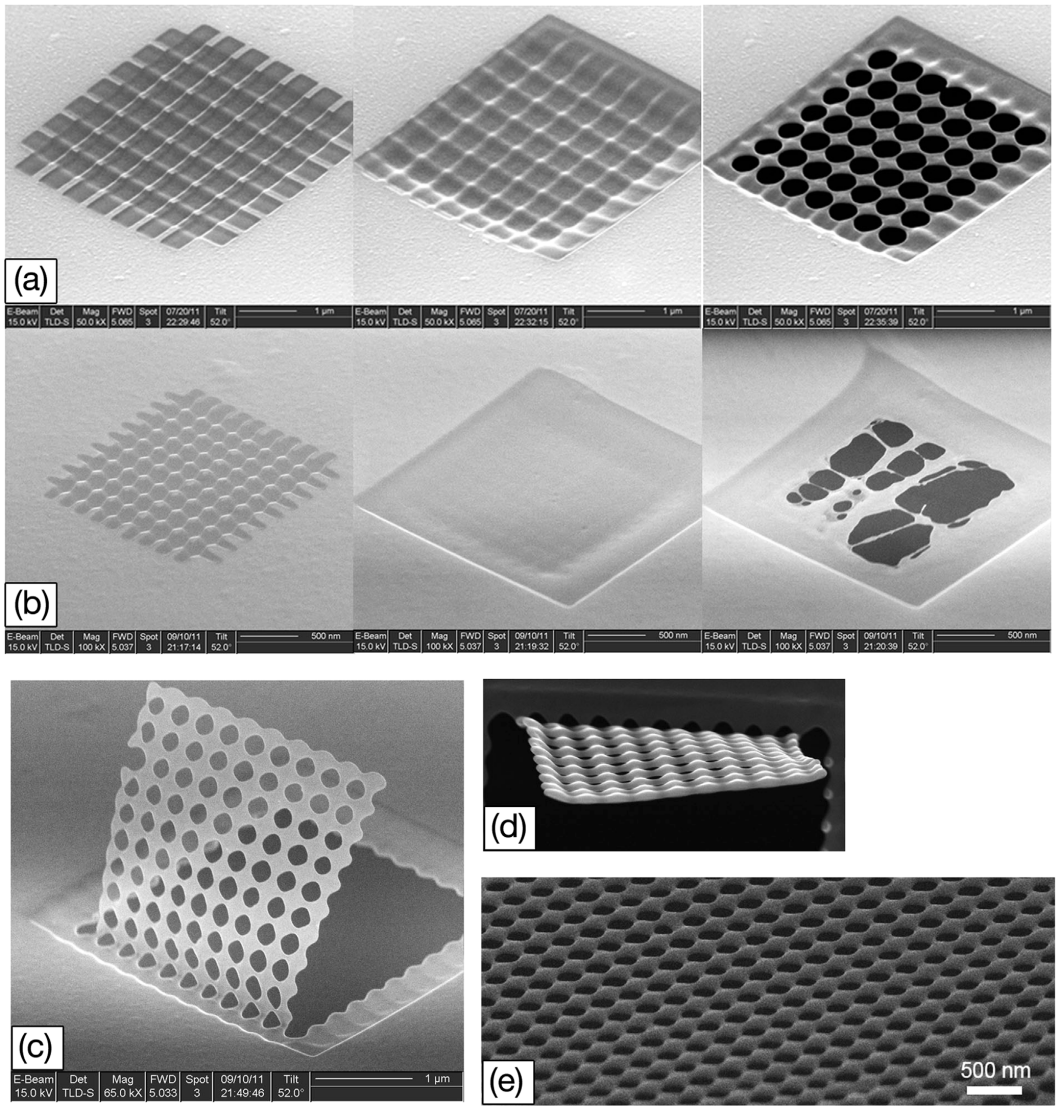
Fabrication of nanopore net using uniform large-area FIB irradiation on suspended membrane with 2D predefined patterns. (a) The 2D trench patterns with a period of 600 nm evolve into a nanopore membrance. (b) The 2D trench patterns with a period less than 200 nm diminishes under FIB bombardment. The breaking-up of membrane does not follow the predefined patterns and is random. (c) Membrane with nanopores was bended up by FIB introduced stress. (d) Top view of the up-bended membrane shows the sinusoid undulation. (e) 52 tilt view of a large-area nanopore membrane fabricated by this method. The average diameter of the pores is about 200 nm.

The mechanism of structure evolution under two-dimensional (2D) predefined patterns is similar with that under one-dimensional (1D) predefined patterns. We employed FIB direct writing to make two groups of periodic trenches, which perpendicularly crossed each other, to compose a 2D predefined pattern on suspended nano-thick films, and then utilized large-area FIB scanning to irradiate the films. We find that the proper 2D predefined patterns could evolve into suspended nanopore nets during the process. With continuous FIB scanning, the pores emerge at the crossed points of the two groups of trenches and enlarge quickly in the suspended films. Nanopore nets are finally formed with in-time stop of the ion irradiation. In our experiments, the periods of the patterns were set to be 600 nm (Figure 6a) and 200 nm (Figure 6b), respectively. The patterns with 600-nm-period lead to nanopore nets while the patterns with 200-nm-period are diminished during the FIB process. Figure 6c shows a nanopore membrane up-folded in-situ via FIB stress-induced deformation^8^. The side view of the membrane clearly demonstrates a 2D sinusoid undulation (Figure 6d). Finally a 5 μm × 5 μm nanopore net on a Si membrane was fabricated, with pore diameter of about 200 nm (Figure 6e).

#### Material and FIB setting factors

**Figure 7:**
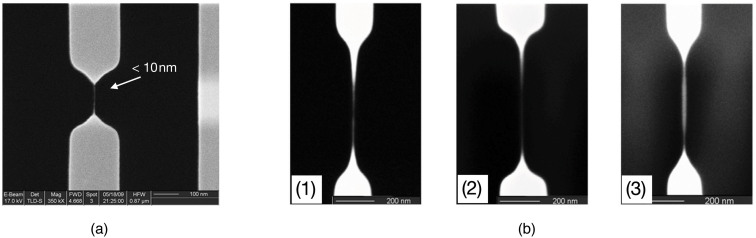
(a) Sub-10 nm nanostring was formed on Si_3_N_4_ cantilever. (b) Comparison experiments to demonstrate the mechanism. For certain material, (b1) and (b2) are the comparison of different ion energy irradiations, which are 15 keV and 30 keV respectively, with the same flux of 3.1 × 10^17^ ion·cm^−2^. (b3) and (b2) are the comparison of different ion flux irradiations, which are 3.1 × 10^17^ ion·cm^−2^ and 1.5 × 10^18^ ion·cm^−2^ respectively, with the same energy of 30 keV.

For the obtained amorphous Si_3_N_4_ and SiO_2_ suspended structures, instability seldom occurs in their morphology evolutions during the FIB irradiation process. The possible reason would be related to the natures of the amorphous materials. As we previously mentioned, structures would be stable if the corresponding materials are of high viscosity, because viscous and even elastic forces rather than capillary force would be dominant during the morphological evolution process. The equivalent viscosity of the materials being irradiated by FIB is determined by both target materials and specific FIB settings. Si_3_N_4_ and SiO_2_ may exhibit much larger equivalent viscosity under ion irradiation with the same FIB settings, probably due to their amorphous natures. Using the material of Si_3_N_4_, nanostrings with large length-diameter ratio as well as diameter less than 10 nm have been successfully fabricated (Figures 7a and 2a).

In order to demonstrate that FIB settings could affect the equivalent viscosity, and, under this condition, Rayleigh-Plateau instability would also occur on Si_3_N_4_ structure evolutions, experiments were carried out on some Si_3_N_4_ nano-cantilevers with different FIB parameters, including ion energy and ion flux. The experimental results are shown in Figure 7b. We find that more skinny threads are fabricated with the irradiation of lower ion energy and flux (comparison between Figure 7b1 and Figure 7b2), while fusiform structures are produced with the irradiation of higher ion energy and flux (comparison between Figure 7b3 and Figure 7b2), which are corresponding to the morphology evolutions of the liquid with lower and higher viscosity respectively^29^. It indicates that materials under ion bombardment by higher energy and flux, which means the more electronic excitation and electron-phonon coupling^32^, will become less “viscous”. Therefore, we can control the shapes of final structures by changing or tuning FIB energy and flux.

#### Multi structure parallel fabrication

**Figure 8:**
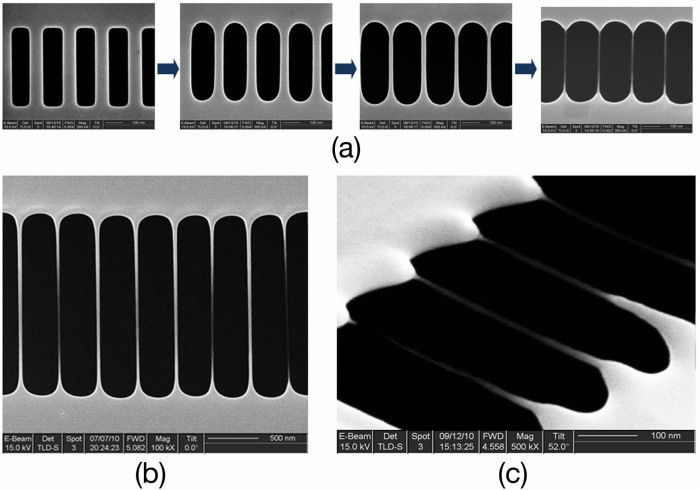
(a) The morphological evolution from multi film cantilevers to multi nanostrings. All the structures are covered simultaneously by FIB large-area irradiation. (b) Eight nanostrings achieved by one-time FIB scanning. (c) Bird's eye view of four nanostrings with diameter less than 10 nm.

By enlarging the FIB irradiation area to cover multi clamped-clamped nano-cantilevers, multi nanostrings can be parallelly fabricated (Figure 8). The detail morphological evolution of the multi structures in the process is shown in Figure 8a, and all the visual area is irradiated by FIB. Under a 34-second FIB irradiation of 30 keV Ga^+^ with a flux of 2.5 × 10^15^ ion/cm^2^·s, multi sub-10 nm nanostrings were successfully produced (Figures 8b and 8c).

## Discussion

In summary, a fabrication method utilizing energetic FIB-induced Rayleigh-Plateau instability has been demonstrated. The resulting structures depend on target materials, predefined patterns, and FIB settings. Based on the method, we have successfully fabricated various suspended structures including nanostrings, fusiform mass structures with nanolinks, nanobead chains and nanopore membranes. The nanostrings and nanolinks have the characteristic dimension of less than 10 nm. The results are consistent with the proposed mechanism. Through adopting proper FIB parameters and scanning patterns, and designing proper initial film structures, more various nanostructures can be achieved. Since the method does not require precisely localized controlling of FIB, it is high likely to transfer this FIB-based method to regular ion process.

## Methods

### FIB Process

The FIB processes were carried out on the FEI Strata 235 Dual Beam System. No beam blanker was used during the processes. The general FIB settings are as follows: overlap = 50%, dwell time = 1 μs, and ion acceleration energy = 30 keV, unless specified in the article. The scanning resolution is 1024 × 884 in the image field, so according to the magnification we used, the step size should be around 8.48 nm. The cantilevers shown in Figures 1–5, 7 and 8 were made by FIB milling with flux of 100 pA. The 1D and 2D predefined patterns shown in Figures 4 and 5 were obtained by FIB milling with flux of 30 pA. The following ion treatment that induces Rayleigh-Plateau instability is in large-area scanning mode. In the process, we choose various fluxes depending on the specific design. The corresponding ion irradiation fluence is calculated based on the specific ion flux and irradiation area.

### Fabrication of suspended films

The SiO_2_ films were firstly achieved by thermal oxidization on Si substrates. On some of the SiO_2_ films, Si_3_N_4_ or poly-Si films were deposited by low pressure chemical vapor deposition (LPCVD). On some others of the SiO_2_ films, Au or Al films were obtained by sputtering. The SiO_2_ membranes were then suspended by wet etching of Si using KOH from the backside of the wafer. The other membranes were suspended by wet etching of Si and then SiO_2_ using KOH and buffered HF (BHF), respectively, from the backside too. The suspended crystal-Si films were obtained by using silicon-on-insulator (SOI) substrates with thin device layers. Like the fabrication process of other films, the crystal-Si device layer membranes were suspended by wet etching of Si handle layers using KOH from backside of the substrates and then SiO_2_ BOX layers using BHF from both sides. The area of suspended film is about 100 μm × 100 μm.
